# Delphi consensus on gene therapy of spinal muscular atrophy with onasemnogene abeparvovec in Germany, Austria and Switzerland – part II – expert based recommendations for surveillance and management of side-effects

**DOI:** 10.1177/22143602261433270

**Published:** 2026-08-27

**Authors:** Andreas Ziegler, Claudia Weiß, Katharina Vill, Matthias Baumann, Günther Bernert, Astrid Blaschek, Astrid Eisenkölbl, Marina Flotats-Bastardas, Johannes Friese, Dorothea Holzwarth, Claudia Ganter, Klaus Goldhahn, Andreas Hahn, Maja von der Hagen, Hans Hartmann, Oswald Hasselmann, Veronka Horber, Ralf A Husain, Sabine Illsinger, David Jacquier, Jessika Johannsen, Cornelia Köhler, Heike Kölbel, Michael Kolodzig, Andrea Klein, Astrid Pechmann, Arpad von Moers, Wolfgang Müller-Felber, Christian Rauscher, Gudrun Schreiber, Oliver Schwartz, Joachim Sproß, Georg M Stettner, Corinna Stoltenburg, Eva Stumpe, Regina Trollmann, Gerd Wiegand, Ekkehard Wilichowski, Ulrike Schara, Janbernd Kirschner

**Affiliations:** 1 Medical Faculty Heidelberg, Center for Pediatric and Adolescent Medicine, Department I, Division of Pediatric Neurology and Metabolic Medicine, 88859 Heidelberg University, Heidelberg, Germany; 2 Department of Pediatric Neurology, 197688 Charité–Universitätsmedizin Berlin, Berlin, Germany; 3 Department of Paediatric Neurology and Developmental Medicine, 9183 Ludwig Maximilian University of Munich (LMU), 27192 Hauner Children's Hospital, Munich, Germany; 4 Division of Pediatric Neurology, Department of Pediatrics I, 155818 Medical University of Innsbruck, Innsbruck, Austria; 5 Department of Pediatrics, Klinik Favoriten, Vienna, Austria; 6 Department of Paediatrics and Adolescent Medicine, Johannes Kepler University Linz, 31197 Kepler University Hospital, Linz, Austria; 7 Department of General Pediatrics and Neonatology, Division of Neuropediatrics, 39072 Saarland University, Homburg Saar, Germany; 8 Department of Child Neurology, 39062 University Hospital Bonn, Bonn, Germany; 9 INTEGRATE ATMP Project, 152528 University Hospital Heidelberg, Medical Faculty Heidelberg, Heidelberg, Germany; 10 Department of Pediatrics and Neuropediatrics, 38179 DRK Klinikum Westend, Berlin, Germany; 11 Department of Child Neurology, 163273 University Hospital, Gießen, Germany; 12 Department of Neuropediatrics, 39063 Medizinische Fakultät Carl Gustav Carus, Technische Universität Dresden, Dresden, Germany; 13 Department of Paediatric Kidney, Liver, Metabolic and Neurological Diseases, 9177 Hannover Medical School, Hannover, Germany; 14 30883 Children’s Hospital Ostschweiz, St Gallen, Switzerland; 15 Department of Paediatric Neurology, 27203 University Children's Hospital, Tübingen, Germany; 16 Department of Neuropediatrics, 39065 Jena University Hospital, Jena, Germany; 17 Pediatric Neurology and Neurorehabilitation Unit, Woman-Mother-Child Department, 30635 Lausanne University Hospital, Lausanne, Switzerland; 18 Department of Pediatrics, 37734 University Medical Center Hamburg-Eppendorf, Hamburg, Germany; 19 Children’s Hospital St Josef, 439861 University Hospital Ruhr-University Bochum, Bochum, Germany; 20 Department of Pediatric Neurology, Centre for Neuromuscular Disorders, Center for Translational Neuro and Behavioral Sciences, 199866 University Duisburg-Essen, Essen, Germany; 21 Deutsche Muskelstiftung, Karlsruhe, Germany; 22 Division of Neuropaediatrics, Development and Rehabilitation, Department of Paediatrics, 27252 Inselspital, Bern University Hospital, University of Bern, Bern, Switzerland; 23 Department of Neuropediatrics and Muscle Disorders, Medical Center – University of Freiburg, Faculty of Medicine, 14879 University of Freiburg, Freiburg, Germany; 24 Department of Pediatrics, 31507 Paracelsus Medical University, Salzburg, Austria; 25 Department of Pediatric Neurology, 60021 Klinikum Kassel, Kassel, Germany; 26 Department of Pediatric Neurology, 237293 Muenster University Hospital, Münster, Germany; 27 German Society for Neuromuscular Diseases e.V. (DGM), Freiburg, Germany; 28 Neuromuscular Center Zürich and Department of Pediatric Neurology, 30995 University Children`s Hospital Zürich, University of Zurich, Zürich, Switzerland; 29 SMA Europe e. V., Freiburg, Germany; 30 Department of Pediatrics, Division of Pediatric Neurology, 9171 Friedrich-Alexander University of Erlangen-Nürnberg, Erlangen, Germany; 31 Department of Pediatrics, Division of Neuropediatrics, 500232 Asklepios Klinik Hamburg Nord-Heidberg, Hamburg, Germany; 32 Department of Pediatrics and Adolescent Medicine, 234891 University Medical Center Göttingen, Göttingen, Germany; 33 INTEGRATE ATMP coordination team, Heidelberg, Germany. Collaborators and members of the INTEGRATE ATMP consortium: Carsten Müller-Tidow, Georg Friedrich Hoffmann, Peter Dreger, Maria-Luisa Schubert, Andreas Ziegler, Stefan Kölker, Thomas Opladen, Ulrike Schara-Schmidt, Nina Rademacher, Bastian von Tresckow, Christian Reinhardt, Marcel Teichert, Veronka Horber, Hendrik Rosewich, Samuel Gröschel, Wolfgang Bethge, Claudia Lengerke, Bärbel Entrich, Claudia Weiß, Angela M. Kaindl, Joanna Schneider, Lisa-Valerie Bitzan, Antonia Busse, Lars Bullinger, Olaf Penack, Maja von der Hagen, Martin Smitka, Josephine Schädlich, Malte von Bonin, Martin Bornhäuser, Raphael Teipel, Astrid Blaschek, Katharina Vill, Wolfgang Müller-Felber, Moritz Tacke, Fabian Hauck, Christoph Klein, Veit Bücklein, Marion Subklewe, Michael von Bergwelt, Regina Trollmann, Fabian Müller, Andreas Mackensen, Dennis Bene, Jessika Johannsen, Ania C. Muntau, Jonas Denecke, Francis Ayuketang Ayuk, Silke Heidenreich, Gesine Bug, Hubert Serve, Julia Riemann, Eva Wagner-Drouet, Beate Hauptrock, Oliver Schwartz, Hélène Guillemot, Heymut Omran, Simon Call, Matthias Stelljes, Georg Lenz, Anna Ossami Saidy, Bertram Glaß, Aleksandar Radujkovic, Sascha Dietrich, Ben-Niklas Bärmann, Friedrich Stölzel, Claudia Baldus, Natalie Schub, Sabine Illsinger, Dieter Haffner, Hans Hartmann, Astrid Pechmann, Janbernd Kirschner, Lutz Hein, Dorothea Holzwarth, Alexandra Klotz, Franziska Busch, Lisa Allenstein, Gabriel Dworschak, Andreas A. Hahn, Bernd A. Neubauer, Marina Flotats-Bastardas, Michael Zemlin, Klaus Goldhahn, Arpad von Moers, Ellen Knierim, Christian Friese, Gudrun Schreiber, Bernd Wilken, Cornelia Köhler, Thomas Lücke, Christoph Hanefeld, Sebahattin Cirak, Antje Westhoff, Kübra Erden, Lisa Benedikter, Sandra Zinke, Gerald Wulf, Wolfram Jung, Justin Hasenkamp.

**Keywords:** Zolgensma, real-world-evidence, advanced therapy medicinal products, ATMP, complications

## Abstract

**Background:**

Systematic protocols for long-term surveillance of children with spinal muscular atrophy treated with onasemnogene abeparvovec are lacking. Together with best practice recommendations for safety monitoring and management, such recommendations should increase patient safety and confidence levels of healthcare providers.

**Objective:**

Based on systematic literature review and evidence grading from part 1, this initiative aims to develop a structured treatment plan applicable across all treatment centers in Germany, Austria and Switzerland. Additionally, it seeks to establish consensus recommendations for the clinical management of safety alerts.

**Methods:**

Part 2 describes the methodology used to formulate Delphi consensus statements, the development of a structured treatment plan for OA treatment, and the anonymous consensus voting process with standardized follow-up in case of disagreement.

**Results:**

A total of 12 consensus statements were developed, addressing diagnostic work-up, safety evaluation, and best practice management of common adverse drug reactions associated with OA gene therapy. All statements achieved >95% consensus in the anonymous Delphi voting. Additionally, two consensus recommendations for handling of positive newborn screening result achieved consensus of 97% and 87%, respectively. A structured treatment plan for gene therapy was consented with 100% agreement, as were standardized recommendations for laboratory testing and a consensus-based algorithm for management of liver transaminase elevations.

**Conclusions:**

Delphi-based expert recommendations, developed in co-creation with patient representatives, provide a framework to minimize complications associated with gene therapy and establish the basis for standardized post-marketing data collections. The methodology used in this Delphi-consensus-group can serve as a blueprint for future gene therapy approvals.

## Introduction

Spinal muscular atrophy 5q13.2 (SMA) in the context of disease-modifying treatments and the growing number of countries implementing SMA in genetic newborn screening was addressed in part 1 of this manuscript. The existence of three approved disease-modifying treatments and the possibility of early initiation of therapy immediately after birth have dramatically changed the course of the disease.^1^ This achievement can lead, in a best-case scenario, to age-appropriate acquisition of motor milestones.^2,3^ However, despite starting treatment very early after birth, a significant number of children still have motor impairments. Onasemnogene abeparvovec (OA) is the first approved gene addition therapy (GAT) for SMA which is now widely used in routine care and beyond the controlled environment of clinical trials. While evidence from clinical trials and a growing body of real-world evidence data supports its use, the overall evidence base remains limited.^3–11^ Due to varying regulatory labels and reimbursement conditions worldwide, the routine use of OA exceeds the age and weight corridors in pivotal trials.^12^ In Germany, the European Medicines Agency (EMA) label allows treatment of children with up to 3 SMN2-copies, without explicit age or weight restrictions.^13^ Switzerland follows the FDA-label and allows treatment of children with up to 3 SMN2 copies up to a maximum age of 2 years. Very recently, the U.S. Food and Drug Administration (FDA) also approved intrathecal gene therapy (onasemnogene abeparvovec-brve) in adult and pediatric patients 2 years of age and older with confirmed mutation in the survival motor neuron 1 (SMN1) gene. Across the D-A-CH region, 31 centers are approved to administer SMA gene therapy, although some centers handle only a small number of cases due to the rarity of the disease.^14^ Therefore, academic collaborative networks and co-creation with patient advocacy groups are key elements to ensure the best possible benefit-risk ratio and minimize serious complications after OA treatment.

The aim of this second part of the consensus process is the development of a structured treatment plan across all treatment centers as well as a consensus on specific recommendations for clinical management of potential safety alerts after OA treatment.

## Methods

Part 2 focuses on the rationale and methodology employed in the Delphi process for formulating the consensus statements and conducting the final anonymous consensus voting, which included at least one representative from all treatment centers and representatives from all relevant national patient organizations as well as SMA Europe as European rooftop organization. Additional methodological details, such as the systematic literature review are described in part 1 of this manuscript.

### Problem area

As the systematic literature review and evidence grading in part 1 revealed that there is still limited knowledge on systematic long-term follow-up and management of possible side effects in children treated with SMA gene therapy, an expert-based Delphi method was used as a well-established method for achieving consensus among experts. A modified real-time Delphi technique with successive virtual Delphi rounds was used to develop a structured treatment plan and systematic protocols for managing potential liver-related side effects. The objective was to facilitate the collection of standardized, homogeneous data sets on treatment effectiveness and pharmacovigilance following OA treatment.^15^

The Delphi panels were integrated into the publicly funded German INTEGRATE-ATMP project,^16^ which applies the same methodology described in parts 1 and 2 of this manuscript across various indications and treatments for all approved gene and cell therapies in Germany, including CAR-T cell therapies. All “vital elements” of the Delphi method (anonymity, iteration, controlled feedback, and statistical stability of consensus) were implemented and the whole process was designed according to published recommendations for appropriateness.^17^ To further demonstrate the methodological quality of the study, the Conducting and Reporting Delphi Studies (CREDES) checklist was used (see supplement).^18^

### Panel composition and size

To minimize pre-selection bias, at least one representative from each SMA gene therapy center across all approved treatment centers in Germany, Austria and Switzerland were invited to participate in the virtual Delphi meetings. Furthermore, representatives from all relevant non-profit patient advocacies (see part 1 of the manuscript) were invited including SMA Europe as a pan-European organization. The panel was further complemented by relevant representatives, including the coordinators of the disease-specific SMArtCARE registry^19^ and three board members of the Swiss-Reg-NMD-registry. The clinical lead of the newborn screening program, and two members of the ATMP task force of the German Society for Pediatrics and Adolescent Medicine (DGKJ) complemented the panel. The panel size was predefined to be within the optimal recommended range of 30–50 panelists.^17^

### Delphi rounds and development of consensus statement

At the first Delphi meeting, participants were divided into three working groups to analyze existing data from the systematic literature review (see Part 1). During the second and third Delphi meetings, consensus statements were developed according to published recommendations.^20^ An optimal statement length of 8–12 words was predefined. Each statement was supported by a brief rationale. As optimal formate requirement for the rationale section a maximum of 150 characters was defined. Rationales were asked support the content of the consensus statement and, if necessary, provide more precise details on the provided recommendations in the statement., Authors should provide existing references including a short summary of content (maximum 10 per statement, summary <200 words) if appropriate. After each Delphi meeting, results of the systematic review and consensus statements were listed in a shared online document in which all participants were invited to provide controlled, anonymous feedback. A discussion protocol for each Delphi meeting was shared with all panelists. All changes and suggestions were discussed iteratively in subsequent meetings. After the third meeting and a thorough discussion of all open points and comments among the panelists, a core author team of three experts developed the anonymous questionnaire based on the comments and the consensus statements and following literature guidelines for survey development.^15^ To ensure methodological rigor, the questionnaire underwent peer review by two additional panelists with extensive scientific expertise in guideline development and in the field of neuromuscular disorders. Following this review, the final version was programmed and distributed to all participants. Data was collected using an online survey administered through LimeSurvey (LimeSurvey GmbH, n.d.).^21^

### Consensus

**Figure 1.**
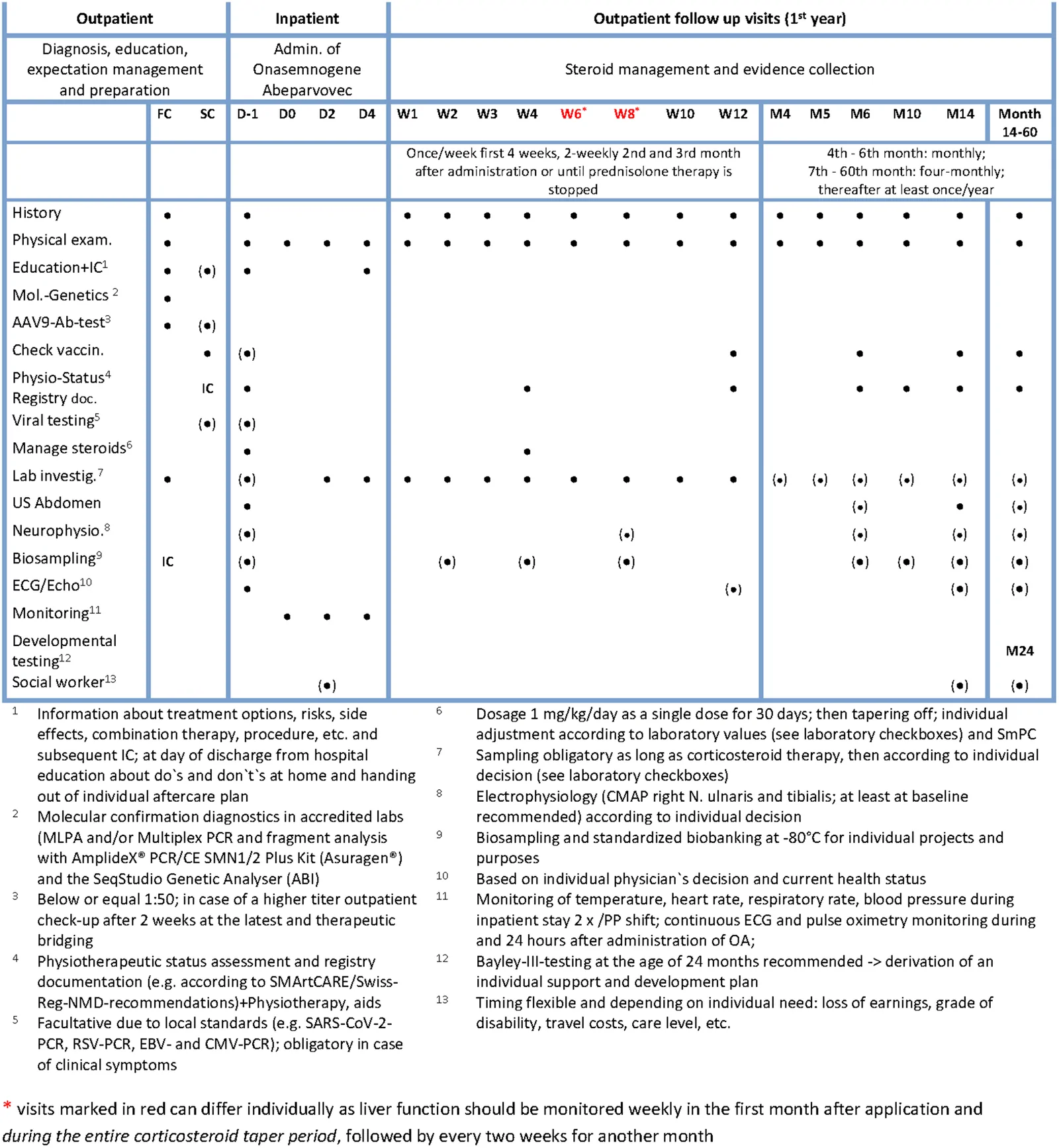
Shows the final version of the structured treatment plan for pre-, inpatient, and aftercare monitoring for children receiving OA in an inpatient setting. The content of the structured treatment plan achieved “strong consensus” (100%, 31 votes). It was recommended that systematic follow-up should take place for at least 15 years after the application of OA, from month 60 after application on at least once per year in the case of a normal motor development and at least twice a year in the case of compromised motor functions at a specialized neuromuscular treatment centre. *Abbreviations:* FC = first contact, SC = second contact, D = day, W = week, M = month, US = ultrasound, AAV9-Ak = Adeno-associated virus 9 antibody, Prednisol.=Prednisolone, IC = Informed Consent, Echo = echocardiography, GOT = glutamate oxaloacetate transferase, GPT = glutamate pyruvate transaminase, RR = blood pressure, IMH = intermediate medical history, SMArtCARE = Longitudinal Data Collection from Patients with Spinal Muscular Atrophy, SmPC = summary of Product Characteristics.

Based on the publication by Simms et al., a 6-point Likert scale was chosen for rating level of consensus.^22^ Following the German guideline recommendations, consensus was predefined as an agreement rate between 75% and 95% among participants, while strong consensus was defined as agreement rate of more than 95% of the panelists.^23^ For the calculation of consensus levels, only responses of level 4 or higher (“agree” and “strongly agree”) were considered. If a participant selected a consensus level of less than or equal to 3 (3 = slightly disagree or lower), an additional comment field was provided to allow specific feedback on the reasons for disagreement and suggestions for improvement. The fourth Delphi meeting was held on January 16^th^, 2023, and the survey results were presented. All anonymous feedback indicating disagreement (levels ≤3) was revisited and discussed among all panelists. The final versions of the consensus statements, structured treatment plan, and recommendations for managing of liver-related side effects were subsequently agreed upon. The potential influence of recently published papers on the evidence level of OA and on the formulation of the consensus statements was thoroughly investigated in the literature update screen and analysis in August 2024 (see Figure 1 in part 1 of the manuscript). It was discussed on a case-by-case basis from the core author team. All panelists agreed in a reinvestigation of the published literature that no further updates need to be undertaken to the existing statements (last assessment August 2025).

### Completion criteria

Within this initiative, a conventional Delphi study design of at least four rounds was predefined. However, the open consensus process described above would have triggered additional Delphi rounds in case of substantial disagreement. Therefore, the primary completion criterion was a substantially high level of consensus of at least >75% among all panelists after extensive discussion and anonymous feedback rounds.

### Stability of the results

According to literature, stability was defined as consistency of responses between successive rounds of our study. The criteria for stopping the Delphi rounds were therefore defined a priori as reaching at least “consensus” or “strong consensus” on all statements, the structured treatment plan, recommendations for laboratory investigations and best-practice management of liver side-effects.^24^

## Results

The results of four consensus statements and anonymous voting on the current evidence concerning the effectiveness of OA treatment are presented in part 1 of this manuscript. Twelve further consensus statements were formulated on diagnostic work-up, safety investigations and best practice management of the most common side effects of gene therapy with OA. In addition, panelists decided to formulate and vote on two additional general statements concerning the management and initiation of treatment in children diagnosed with SMA through newborn screening. Table 1 summarizes all 14 consensus statements and displays the distribution of consensus scores as well as the final consensus percentages reached within the D-A-CH region. All 12 consensus statements concerning safety of OA treatment reached “strong consensus”, with 10 out of 12 reaching 100% agreement. The two general statements on the approach following positive newborn screening for SMA reached strong consensus (97%; immediate treatment) and consensus (87%; choice of treatment and bridging therapy). The only statement that did not reach “strong consensus” was statement 18 (see below). Two panelists scored “slightly disagree” and “disagree”, not due to the statement itself, but due to concerns regarding the rationale. As a result, during Delphi meeting 4 it was decided to slightly revise the final text of the rationale, but not the statement itself. Table 2 shows the distribution of consensus results for the structured treatment plan, recommendations for laboratory monitoring of children treated with OA, and best-practice management of liver side effects, all of which achieved “strong consensus”.

**Table 1.** Summarizes the results from anonymous Delphi consensus voting concerning safety of OA therapy (statement 5–16) and general aspects (statement 17 and 18). Consensus was predefined as an agreement rate between 75% and 95% among participants, while strong consensus was defined as agreement rate of more than 95% of the panelists. For the calculation of consensus levels, only responses of level 4 or higher (“agree” and “strongly agree”) were considered. If a participant selected a consensus level of less than or equal to 3 (3 = slightly disagree or lower), additional comments were provided which are included and marked with stars*1 and *2.

Group	Statement-number	Consensus statement	Answer options						Votes	Consensus of 5 and 6
			1	2	3	4	5	6		
			%	%	%	%	%	%		
Safety	5	Prior to gene therapy, baseline diagnostic work-up is required following expert-panel-recommendations and the summary of product characteristics (SmPC).	0	0	0	0	19.35	80.65	31	100
	6	Elevations of transaminases (>2× ULN) or cholestasis parameters require additional work up before gene therapy.	0	0	0	0	29.03	70.97	31	100
	7	The vaccination scheme should be reviewed and possibly adjusted before and after gene therapy.	0	0	0	3.23	29.03	67.74	31	96.77
	8	Gene therapy must be administered in experienced hospitals and inpatient monitoring should be provided for at least 72 h.	0	0	0	3.23	19.35	77.42	31	96.77
	9	The coordination and responsibility of follow-up lies with the center for gene therapy.	0	0	0	0	29.03	70.97	31	100
	10	Clinically relevant blood count abnormalities and transaminase elevations should be monitored until normalization.	0	0	0	0	22.58	77.42	31	100
	11	Children >8 months of age at time of therapy should receive at least 8 weeks of immunosuppressive treatment.	0	0	0	0	51.61	48.39	31	100
	12	Prednisolone should only be tapered after 4 (8) weeks when transaminases are <2xULN.	0	0	0	0	48.39	51.61	31	100
	13	Therapy for transaminase elevation should be based on magnitude, dynamics, and clinical symptoms.	0	0	0	0	35.48	64.52	31	100
	14	In case of suggestive clinical symptoms and/or laboratory abnormalities (e.g., thrombocytopenia < 20,000/nL), diagnostic investigation for thrombotic microangiopathy (TMA) should be performed without delay.	0	0	0	0	9.68	90.32	31	100
	15	Already in case of clinical and/or laboratory suspicion of TMA, steroid dose should be increased.	0	0	0	0	25.81	74.19	31	100
	16	Hemophagocytic lymphohistiocytosis (HLH) could be a complication of gene therapy.	0	0	0	0	51.61	48.39	31	100
General	17	If clinical and/or electrophysiologic symptoms of SMA are present after positive newborn screening for SMA, immediate initiation of treatment is indicated.	0	0	0	3.23	25.81	70.97	31	96.78
	18	In case of high-risk constellations after positive SMA newborn screening and a decision for bridging therapy, different modes and onset of action should be considered in the choice of the drug.	0	3.23^a^	3.23^b^	6.45	29.03	58.06	31	87.09

^a^ Agree with the statement, but not with all parts of the rationale. What do we really know about the time to restore deficient SMN protein levels in motoneurons for the three drugs? This is complex and data is not readily available.

^b^ Genetic confirmation diagnostics should be available within 3–5 business days. In a newborn with symptoms of the disease already present at the time of birth (high-risk constellation. newborn child with positive SMA-screening AND clinical symptoms (when number of SMN2-copies is still unknown)), the presence of 1 SMN2 copy is also possible. In this case, palliative care must also be discussed within the framework of shared decision making with the parents before starting “off-label” drug treatment.

**Table 2.** Shows results of the anonymous Delphi votings on the structured treatment plan pre- and after administration of OA shown in Figure 1, recommendations for laboratory investigations (shown in Figures 2 and 3**)** and a structured management of transaminase elevations after OA treatment, shown in Figure 4. If a participant selected a consensus level of less than or equal to 3 (3 = slightly disagree or lower), additional comments were provided which are included and marked with star *1.

Group	Consensus statement	Answer options						Votes	Consensus of 5 and 6
		1	2	3	4	5	6		
		%	%	%	%	%	%		
General	Please review the following structured treatment plan for gene therapy pre-, inpatient- and aftercare. Please note that the plan already includes the updated recommendations of the Summary of Product Characteristics (SmPC) from March 14th, 2023 (marked in red).	0	0	0	0	38.71	61.29		100
	Please review the following recommendations for laboratory investigations pre- and post-application of Onasemnogene Abeparvovec carefully. All comments of the consensus group were included in the final document.	0	0	0	0	38.71	61.29		100
	Please review the following extended recommendations in the event of a serious adverse drug reaction (acute liver reaction, thrombotic microangiopathy).	0	0	3.23^a^	0	32.26	64.52		96,78
	Please review the following algorithm for the management of acute, subacute and secondary increase of transaminases. The final graph includes all the expert recommendations and comments of the consortium	0	0	0	0	22.58	77.42		100

^a^ I would clarify and list the exact lab controls recommended in add: for TMA.

**Figure 2.**
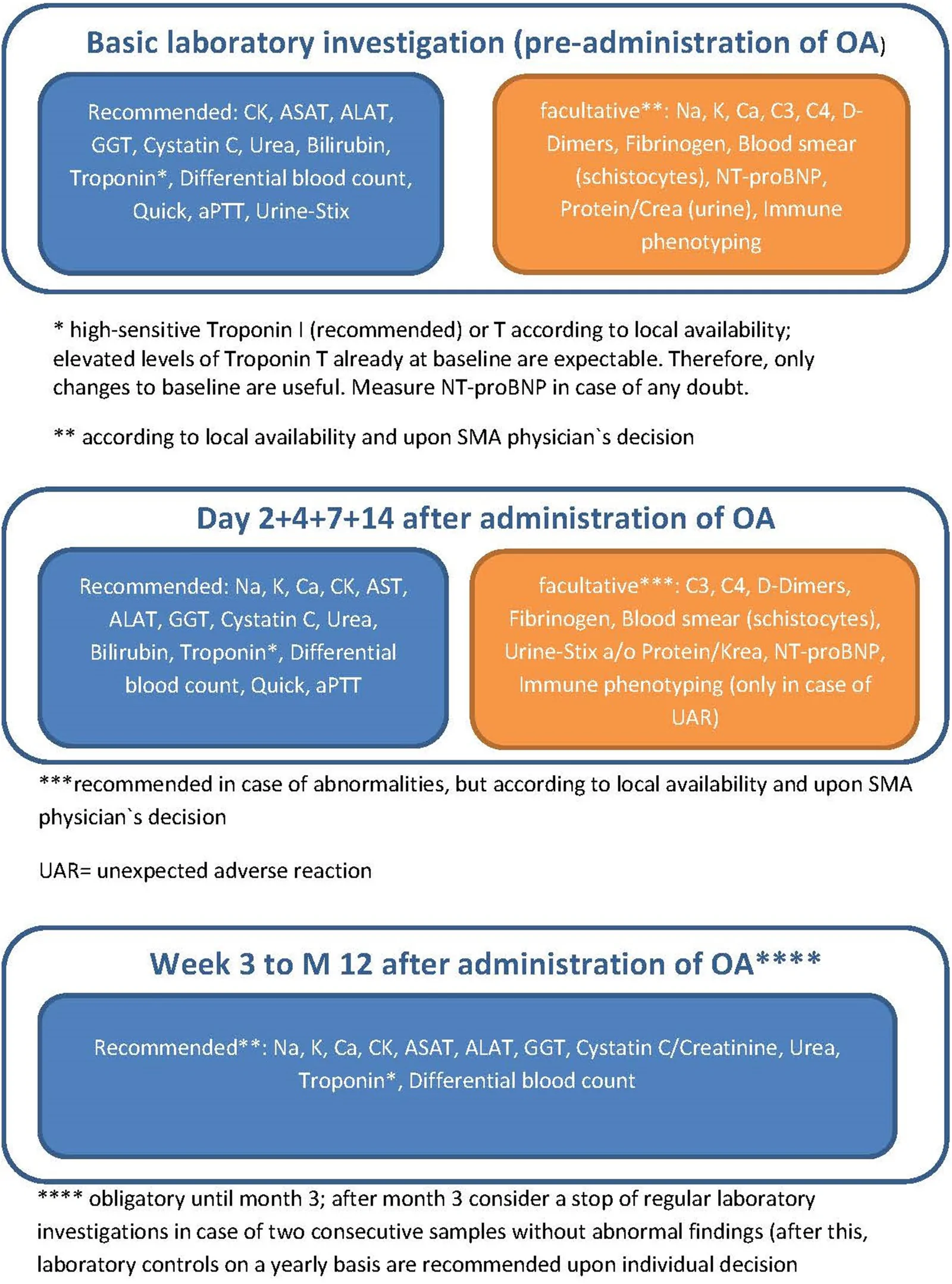
Summarizes all recommendations for laboratory investigations and time points for pre- and post-application of OA in a routine setting (“strong consensus”, 100%, 31 votes).

**Figure 3.**
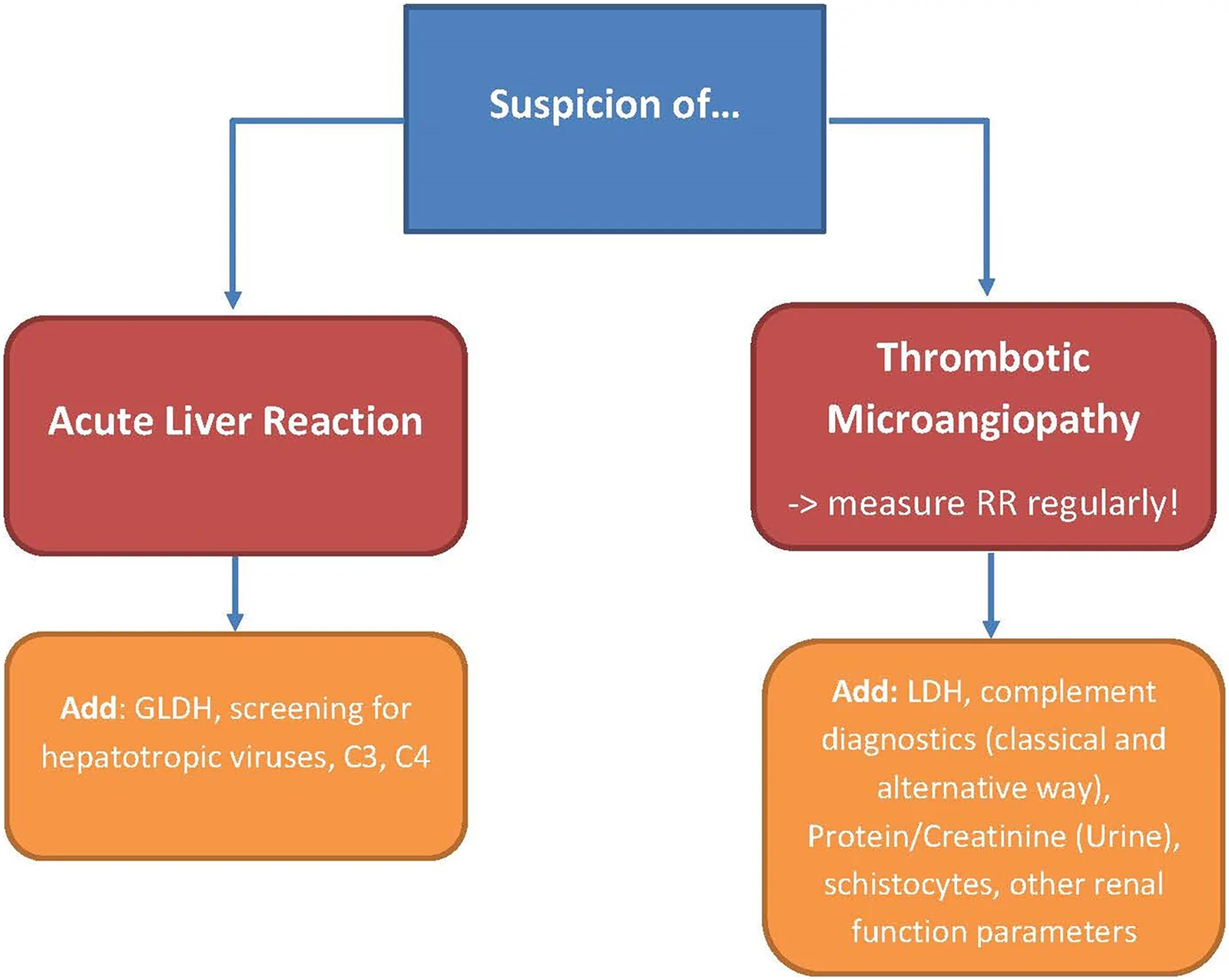
Presents a more comprehensive and detailed laboratory schedule in the event of suspicion of either an acute liver reaction or thrombotic microangiopathy (TMA) with “strong consensus” among all panelists (96,78%, 31 votes). To achieve a structured and systematic management of potential increases of liver transaminases after OA treatment, the Delphi Panel agreed to develop an algorithm based on treatment experience and existing literature from clinical trials and post-marketing observational studies. Therefore, management of elevated transaminases was divided according to their timely appearance into acute, subacute and secondary increase of transaminases after a first tapering of oral steroids.

The following rationales and literature sources were incorporated into the Delphi consensus voting:

**Table:** 

**Consensus statement 5 (safety):** prior to gene therapy, baseline diagnostic work-up is required following expert-panel-recommendations and the summary of product characteristics (SmPC). **(“Strong consensus”, 100%, 31 votes)**
*Rationale:* Prior to gene therapy, 5q SMA must be genetically confirmed, and the *SMN2* copy number must be determined. Additionally, AAV9 titers, abdominal sonography, echocardiography, ECG, blood count, and renal and liver function values have to be assessed. Cranial ultrasound may also be performed in young infants as an additional evaluation. The objective of this expert panel was to exactly define the diagnostic work-up based on real-world observations (see structured treatment plan for details).
*Literature sources:* Most of the discussed baseline investigations are already part of the current summary of product characteristics for OA.^13^ As a summary of larger post-marketing data-collections from different countries, the diagnostic work-up was extended to take account of possible additional risks, such as cardiotoxicity, potential hepatobiliary comorbidities, and baseline investigation of renal function.^25–39^ Laboratory investigations will be discussed separately.

**Table:** 

**Consensus statement 6 (safety):** elevations of transaminases (>2× ULN) or cholestasis parameters require additional work up before gene therapy. **(“Strong consensus”, 100%, 31 votes)**
*Rationale:* Because gene therapy is associated with the risk of therapy-associated hepatopathy and involves relevant immunosuppression, pre-existing hepatopathy potentially increases the risk of severe liver side effects. Patients with elevated transaminases and/or bilirubin levels (except due to neonatal jaundice) or other signs of liver dysfunction should only be treated with onasemnogene abeparvovec after an intensive diagnostic work-up and in exceptional cases. Treatment should not be delayed in these cases. In case of doubt, nusinersen or risdiplam should be considered as alternative SMN upregulating treatments.
*Literature sources:* In patients with SMA, liver function can be affected within the multisystemic nature of the disease.^40^ Furthermore, other comorbidities such as hepatotropic viral infections or other congenital or acquired diseases of the hepatobiliary tract should be extensively investigated in case of any laboratory abnormalities in the baseline screening before OA therapy.^8,9,26,29,32,37^ Relative or absolute contraindications for OA therapy should result in the decision on an alternative treatment option according to the SmPC.^13^

**Table:** 

**Consensus statement 7 (safety):** the vaccination scheme should be reviewed and, if necessary, adjusted both before and after gene therapy. **(“Strong consensus”, 96,77%, 31 votes)**
*Rationale:* The immunosuppressive effect of steroid therapy can be significant depending on dose regimen and duration. The risk of incomplete vaccination response under steroid therapy must be carefully balanced against delayed immunization. Vaccination prior to gene therapy may increase the risk of hepatopathy due to nonspecific activation of the immune system. Therefore, no vaccinations should be given within (2-) 4 weeks before gene therapy (live-attenuated or inactivated vaccines). Ideally, after (2-) 4 weeks following steroid therapy weaning, the vaccination schedule should be completed with inactivated vaccines, while live-attenuated vaccines should be administered no sooner than 2 months after steroid therapy cessation (including Rota-Virus vaccination). An exception can be made, particularly in newborns without any vaccination before therapy: If steroid therapy is still ongoing for more than 4 weeks after GT, these infants may receive inactivated vaccines based on an individualized decision. Beyond the neonatal period, basic immunization should, whenever possible, be completed before gene therapy, in accordance with national vaccination recommendations. Furthermore, symptomatic SMA children with proximal weakness and/or an increased risk of pulmonary infections should receive respiratory syncytial virus (RSV) prophylaxis during the first two years of life.
Comment: in the meantime, after the consensus voting took place, German guidelines for RSV-prophylaxis have changed. The German Standing Committee on Vaccination (STIKO) now recommends a single RSV prophylaxis with Nirsevimab for all newborns and infants in their first RSV season.
*Literature sources:* Vaccination recommendations for SMA patients before and after gene therapy are currently only available from small expert groups^32,41^ and from the Polish Vaccinology Association.^42^ The consensus statement is based on general recommendations for patients with SMA^43^ for vaccinations in the context of immunomodulatory therapies and a case report of thrombotic microangiopathy (TMA) after vaccination.^30,44,45^ Recommendations regarding passive RSV immunization are based on international recommendations.^46^

**Table:** 

**Consensus statement 8 (safety):** gene therapy must be administered in experienced hospitals and inpatient monitoring should be provided for at least 72 h after the treatment. **(“Strong consensus”, 100%, 31 votes)**
*Rationale:* Clinical complications (anaphylaxis, fever, vomiting, hepatopathy, TMA) frequently manifest within the initial week after gene therapy. There is at least some evidence that the “window of mandatory therapeutic intervention” in case of a severe side effect after gene therapy is less than 24 h. As early signs of acute liver failure and/or TMA might be missed without regular monitoring of clinical status, vital signs and supplementary laboratory investigations, inpatient monitoring is strongly recommended. Exceptions might be considered in cases where very close and daily monitoring can be guaranteed in experienced treatment centers.
*Literature sources*:^3,11,28,39,45,47–50^

**Table:** 

**Consensus statement 9 (safety):** the coordination and responsibility of follow-up care rest with the center for gene therapy. **(“Strong consensus”, 100%, 31 votes)**
*Rationale:* Depending on the course and age, the frequency of follow-up appointments should generally be weekly in the first month and upon decision of the individual physician during the entire corticosteroid taper period if necessary, then biweekly until week 12, then monthly until month 6, and then no less than every four months (see structured treatment plan, Figure 1).
*Literature sources*:^4,10–13,25,27,31,39,51–57^

**Table:** 

**Consensus statement 10 (safety):** clinically relevant blood count abnormalities and transaminase elevations should be monitored until normalization. **(“Strong consensus”, 100%, 31 votes)**
*Rationale:* Platelet decrease in combination with endothelial activation and signs of hemolysis can lead to one of the most severe side effects of OA treatment, thrombotic microangiopathy (TMA). Laboratory abnormalities may precede clinical signs and must therefore be closely monitored. Another severe side effect is liver dysfunction which can in severe and untreated cases lead to liver failure and death. Thrombocytopenia and transaminase elevation are therefore important laboratory parameters that should be recorded regularly. The expert panel has therefore developed comprehensive recommendations, with a particular focus on the effective monitoring and management of transaminase elevations.
*Literature sources:* see structured treatment plan and statement 9.

**Table:** 

**Consensus statement 11 (safety):** children older than 8 months at time of gene therapy should receive a minimum of 8 weeks of immunosuppressive treatment. **(“Strong consensus”, 100%, 31 votes)**
*Rationale:* It was shown in recent real-world observations that children older than 8 months at time of OA treatment who receive prednisolone therapy with 1 mg/kg/day for only four weeks are at higher risk of second rise in transaminases if weaned too early. Prophylactic therapy with 1 mg/kg/day prednisolone should therefore be continued for at least 8 weeks in older children (>8 months) to potentially prevent a second rise in transaminases.
*Literature sources:* Mean duration of steroid treatment across all larger real-world-observations varied, but in all reported cases of children older than 8 months, it exceeded an average of 8 weeks. Steroid regimens should be used with caution, particularly in newborns, and tapering of the steroids should be initiated as soon as possible. In Newborns earlier tapering of steroids (<4 weeks) can be considered in cases with no signs of side effects and under close monitoring.^3,7,9,10,11,27,51^

**Table:** 

**Consensus statement 12 (safety):** prednisolone should only be tapered after 4 (8) weeks when transaminases are <2xULN. **(“Strong consensus”, 100%, 31 votes)**
*Rationale:* Following SmPC recommendations and real-world evidence, prednisolone should not be discontinued until transaminases have dropped <2xULN. Tapering should be done according to in-house standards.
*Literature sources*:^13,26,28^

**Table:** 

**Figure 4.**
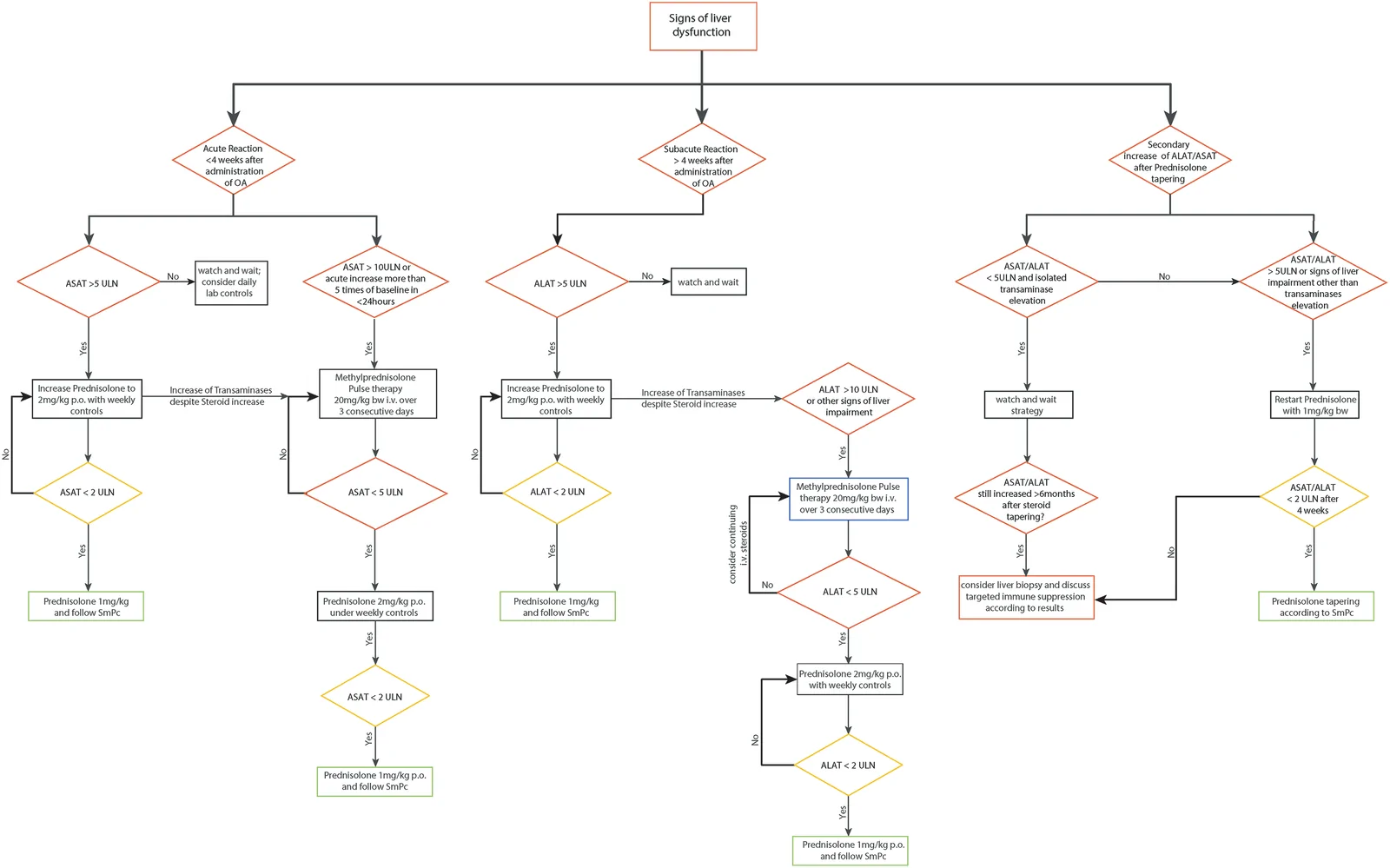
Shows the expert-based algorithm for a structured management of acute, subacute or secondary increases of liver transaminases, which again reached strong consensus (100%, 31 votes) among all panelists.

**Consensus statement 13 (safety):** therapy for transaminase elevation should be based on magnitude, dynamics, and clinical symptoms. **(“Strong consensus”, 100%, 31 votes)**
*Rationale:* If transaminases are elevated >5xULN and the patient is in good general condition, an increase in the prednisolone dose to 2 mg/kg/day should be considered. Acute liver failure is an emergency situation and requires immediate action including hospital admission. Methylprednisolone pulse therapy at 20–30 mg/kg/day on three consecutive days must be considered. The expert panel has therefore made dedicated recommendations, also in case of a secondary increase of transaminases after an initial tapering (see below).
*Literature sources:* There are no controlled, protocol-based intervention studies for the systematic management of transaminase elevations in the context of OA treatment. Therefore, the Delphi panel has developed a systematic algorithm that will serve as basis of future systematic management of this possible adverse drug reaction (ADR); see Figure 4.^3,4,8,11,38,39,58^ The algorithm will be reassessed by the Delphi panel every 6–12 months and potentially revised, in accordance with the evolving body of evidence.

**Table:** 

**Consensus statement 14 (safety):** in case of suggestive clinical symptoms and/or laboratory abnormalities (e.g., thrombocytopenia < 20,000/nL or rapid decline of platelet counts), diagnostic investigation for TMA should be performed without delay. **(“Strong consensus”, 100%, 31 votes)**
*Rationale:* TMA is a rare but life-threatening complication that occurs independently of age, typically in the first two weeks following gene therapy. Clinical warning signs of TMA include oliguria, proteinuria, hematuria, hypertension, and edema. Hemolysis parameters, complement factors (C3, C4), and fragmentocytes complete the diagnostic workup (see laboratory checkboxes).
*Literature sources*:^30,39,45,47,50^

**Table:** 

**Consensus statement 15 (safety):** in case of clinical and/or laboratory suspicion of TMA, steroid dose should be increased. **(“Strong consensus”, 100%, 31 votes)**
*Rationale:* TMA is an emergency situation and requires immediate action. Treatment options in case of early signs (rapid decline in platelet counts, complement activation, signs of anemia and/or hemolysis, newly positive schistocytes in peripheral blood smear) include a proactive increase of prednisolone dose up to 2 mg/kg/day or methylprednisolone pulse therapy. In case of manifest TMA, eculizumab or hemofiltration / dialysis should be considered.
*Literature sources:* see consensus statement 14.

**Table:** 

**Consensus statement 16 (safety):** hemophagocytic lymphohistiocytosis (HLH) could be a rare but serious complication of gene therapy. **(“Strong consensus”, 100%, 31 votes)**
*Rationale:* HLH has been reported once in association with gene therapy for SMA. Symptoms and signs include cytopenias, organomegaly, inflammation, and skin rash. Diagnosis and therapy should follow hemato-oncologic recommendations.
*Literature sources*:^59^

**Table:** 

**Consensus statement 17 (general):** if clinical and/or electrophysiologic signs of SMA are present following a positive newborn screening, immediate treatment initiation is indicated. **(“Strong consensus”, 96,78%, 31 votes)**
*Rationale:* In a significant proportion of children with SMA, particularly those with one or two SMN2 copies, neurodegeneration begins perinatally. Evidence from SMA newborn screening pilot projects indicates that a considerable proportion of children already experience motor regression within the first four weeks of life, especially if clinical and/or electrophysiological signs of disease onset are present at first clinical assessment. Early initiation of therapy is the key factor for therapeutic success. Preparatory assessments and logistical steps for gene therapy should not result in a delay of treatment initiation. In such “risk constellations” bridging therapy with a readily available drug should be considered.
*Literature sources*:^3,54,60–63^

**Table:** 

**Consensus statement 18 (general):** in case of high-risk constellations after positive SMA newborn screening and a decision for bridging therapy, different modes and onset of action should be considered in the choice of the drug. **(“Consensus”, 87,09%, 31 votes).**
*Rationale:* High-risk-constellations are defined as follows: a newborn child with positive SMA-screening AND clinical symptoms (when number of *SMN2* copies is still unknown) OR a newborn child with positive SMA-screening AND 2 *SMN2* copies. Active disease progression of SMA, which is triggered by a critical deficiency of SMN protein in motor neurons, can occur already in the third trimester of pregnancy and immediately after birth in high-risk constellations. Damaged motoneurons are completely incapable or only to a very limited extent capable of regeneration. On the basis of the observations made so far and the supporting data, the working group has concluded that the initiation of therapy at the earliest timepoint possible (even a few days earlier), could have a significant impact on to the outcome in this patient group. This is the justifying reason for an immediate start of therapy on the basis of the above-mentioned conditions. In high-risk-constellations initiation of therapy can even be justified based on a positive DBS screening result (ideally including determination of *SMN2* copy number) and should be re-evaluated after confirmation diagnostics. The two substances, Nusinersen and Risdiplam, are generally applicable in symptomatic patients even prior to documented knowledge of the SMN2 copy number.
**Background and existing literature:** The following discussion will address the potential advantages and disadvantages, as well as the cost-effectiveness of the two approved mRNA therapies for SMA, Nusinersen and Risdiplam, in bridging therapy. For all three disease modifying treatments an increase in SMN-protein levels has been observed; however, the impact of this increase on the function and survival of motor neurons remains to be elucidated. The expert panel has therefore derived practical guiding recommendations.
**1. Nusinersen**
*Advantages:*
This drug has been known the longest, has been used the most to date, and thus has the best-known benefit-risk profile, which can be considered favourable overall. It is approved for the treatment of SMA patients of all ages and with all *SMN2* copy numbers. The use of Nusinersen as bridging therapy is thus certainly in-label and justifiable in the presence of 5qSMA. Intrathecal use is invasive, but injection ensures application to the target organ. Time until first start of treatment lies only in the range of 1–3 working days in most experienced centers.
*Possible disadvantages:*
The pharmacokinetics of Nusinersen might not be fast enough to immediately restore deficient SMN-protein-levels in motoneurons.^64,65^ Additionally, administration is intrathecal and thus invasive. However, in the neonatal period, sedation is not necessary in the majority of patients. Nusinersen has not been tested in healthy humans. Thus, in the very unlikely but principally possible event of false positive newborn screening result, the effect on individuals not affected by SMA remains unknown.
*Cost-effectiveness:*
The single dose of Nusinersen costs around 80,000 EUR. Thus, the cost of therapy as a bridging drug without subsequent continuous therapy is 80,000–160,000 EUR for 1–2 injections until a potential switch to OA.
*Recommendation for use:*
Nusinersen can be used immediately as a bridging or one-and-only-therapy-option in clinically or electrophysiologically symptomatic newborns with positive SMA diagnosis.
**2. Risdiplam:**
*Advantages:*
Risdiplam is administered orally and must be taken once daily. The risk and side effect profile to date is considered favourable, but safety evidence is still limited. Following the initiation of therapy, a rapid increase in SMN-protein levels has been observed.^66^
*Disadvantages:*
At the time of the Delphi consensus voting, Risdiplam was only approved for children older than 2 months of age, therefore the use in neonates after positive SMA newborn screening was “off-label”. In the meantime, the label of Risdiplam has been extended and newborn children can also be treated with Risdiplam directly after birth. The label covers symptomatic patients with 5q-SMA and those with up to 4 *SMN2* copies. Administration is non-invasive, but cannot be fully ensured in individual cases when the neonate “spits up”. The substance is not commonly stocked in treatment centres, so the centre must make advance arrangements to ensure timely initiation of therapy. Risdiplam has not been tested in healthy humans. Thus, in the very unlikely but theoretically possible event of false positive newborn screening result, the effect on individuals not affected by SMA remains unknown.
*Cost-effectiveness:*
The smallest amount of drug than can be prescribed currently costs approximately 10,000 EUR. Bridging therapy for 1–3 weeks until a possible switch to onasemnogene abeparvovec therefore shows high cost-effectiveness. The possibility of aliquoting could be negotiated with the manufacturing company, which would further reduce costs.
*Recommendation for use:*
At the time of the Delphi consensus voting Risdiplam had not received approval in Europe for its use in newborns with SMA diagnosis in newborn screening. An off-label-use could therefore not be generally recommended in this age-group. Subsequent to the Delphi consensus, Risdiplam has been approved for use in newborns diagnosed with SMA through neonatal screening. Consequently, the expert panel considers Risdiplam as a bridging or one-and-only-therapy-option in clinically or electrophysiologically symptomatic newborns with positive SMA diagnosis.
*Literature sources*:^65–67^

## Discussion

One-time GAT, such as OA, which is licensed in large parts of the world, has the potential to substantially change SMA disease courses, particularly if it is administered immediately after birth during the asymptomatic stage. However, a systematic analysis and evidence grading of the existing literature in part 1 of this manuscript revealed that expert-based recommendations and structured knowledge transfer among treatment centers with varying experience and patient numbers are crucial to ensure optimal treatment safety and to establish systematic follow-up algorithms and best-practice protocols for managing potential safety concerns.

This second part of the manuscript delineates 14 expert-based recommendations for safety monitoring and general care of children treated with OA across all SMA gene therapy centers in Germany, Austria and Switzerland. Notably, all recommendations met the predefined stability criteria within the Delphi-process, attaining either “strong consensus” or “consensus”. This underscores the feasibility and robustness of the Delphi method in this initiative, demonstrating its potential as a blueprint for other indications in the context of emerging gene-modifying therapies. Rare instances of “slight disagreement” with the statements prompted additional comment, which were addressed in a final Delphi meeting but did not necessitate revisions of the consensus statements. The highly standardized methodological process was also utilized to consent on a structured treatment and follow-up schedule for children treated with OA and to harmonize laboratory investigations and collection time points across all centers. The resulting guidelines now serve as basis for national quality assurance guidelines for the administration of OA, at least in Germany. It is important to note that not all recommendations may be generalizable to other parts of the world, due to locally different healthcare system structures.

A key aspect of this process was the systematic and early involvement of patient advocacy groups along with representatives of the SMA registry, the newborn screening program, and the experts in Pediatric Neurology including representatives of the German Society of Neuropediatrics (GNP). This approach aims to minimize the burden on families, who must attend frequent outpatient visits, particularly during the first year after the administration of OA. However, adherence to the structured follow-up algorithm established in this Delphi initiative will require substantial additional resources in neuromuscular centers. Consequently, healthcare infrastructures must be adapted to accommodate the unique demands of new therapies, ensuring long-term follow-up and systematic documentation of therapy outcomes for at least 15 years in specialized neuromuscular treatment centers, linked to central disease- or method-specific registries. Therapy outcome observations for gene therapy have to include long-term safety-observations due to potential risk of tumorigenicity due to a theoretically possible integration of AAV vector DNA into the human genome.

A harmonized and structured treatment plan for new therapies across all centers, including systematic post-marketing data collection in academic registries, can also form the basis for effort estimation and reimbursement models by national healthcare authorities. A cost-covering reimbursement of expenses and resources is essential to meet the growing demand for specialized personnel required for the implementation of advanced therapies in the future.

Another critical goal of this initiative was to harmonize recommendations for the managing of potential treatment-related side effects, considering the diverse clinical experiences and patient volumes across the centers in Germany, Austria and Switzerland. The resulting diagnostic and therapeutic recommendations are highly specific and designed to enhance patient safety. However, these recommendations must be continuously updated as more children receive treatment worldwide and our understanding of the immune responses to the AAV9 vector, the transgene, and the resulting SMN protein levels evolves. A standardized definition, severity grading, and management strategy for potential side effects will help us to determine whether our current understanding and approach to adverse drug reactions to OA is appropriate or whether adjustments are required over time.

In conclusion, the Delphi method and structured consensus process outlined in this two-part manuscript proved highly effective in establishing robust, consensus-based recommendations for SMA gene therapy. The method will also be used for other approved ATMPs in Germany, serving as a systematic blueprint for future gene and cell therapy approvals. Ideally, Delphi-based recommendations should be published alongside the approval of a new gene or cell therapy. Expanding these methodologies to international academic collaborations – beyond just a few countries – would be highly beneficial.^68^ This would facilitate enhanced patient safety, promote systematic data collection and enable comparability of real-world evidence across different registries and treatment settings worldwide.

## Supplemental Material

sj-docx-1-jnd-10.1177_22143602261433270 - Supplemental material for Delphi consensus on gene therapy of spinal muscular atrophy with onasemnogene abeparvovec in Germany, Austria and Switzerland – part II – expert based recommendations for surveillance and management of side-effects Supplemental material, sj-docx-1-jnd-10.1177_22143602261433270 for Delphi consensus on gene therapy of spinal muscular atrophy with onasemnogene abeparvovec in Germany, Austria and Switzerland – part II – expert based recommendations for surveillance and management of side-effects by Andreas Ziegler, Claudia Weiß, Katharina Vill, Matthias Baumann, Günther Bernert, Astrid Blaschek, Astrid Eisenkölbl, Marina Flotats-Bastardas, Johannes Friese, Dorothea Holzwarth, Claudia Ganter, Klaus Goldhahn, Andreas Hahn, Maja von der Hagen, Hans Hartmann, Oswald Hasselmann, Veronka Horber, Ralf A Husain, Sabine Illsinger, David Jacquier, Jessika Johannsen, Cornelia Köhler, Heike Kölbel, Michael Kolodzig, Andrea Klein, Astrid Pechmann, Arpad von Moers, Wolfgang Müller-Felber, Christian Rauscher, Gudrun Schreiber, Oliver Schwartz, Joachim Sproß, Georg M Stettner, Corinna Stoltenburg, Eva Stumpe, Regina Trollmann, Gerd Wiegand, Ekkehard Wilichowski, Ulrike Schara, Janbernd Kirschner and collaborators and members of the INTEGRATE ATMP consortium in Journal of Neuromuscular Diseases
