# Supplementary material for: Delphi consensus on gene therapy of spinal muscular atrophy with onasemnogene abeparvovec in Germany, Austria and Switzerland – part II – expert based recommendations for surveillance and management of side-effects
Source: J Neuromuscul Dis. 2026 Aug 27:22143602261433270. Online ahead of print. doi: 10.1177/22143602261433270 (PMC13522257; doi:10.1177/22143602261433270)
Supplement: Supplementary file 1 [file sj-docx-1-jnd-10.1177_22143602261433270.docx]

| **Section/Topic** | **Item #** | **Recommendation** | **Reported on page #** |
| --- | --- | --- | --- |
| **Purpose and rationale** | 1 | The purpose of the study should be clearly defined and demonstrate the appropriateness of the use of the Delphi technique as a method to achieve the research aim. A rationale for the choice of the Delphi technique as the most suitable method needs to be provided | 8-9 |
| **Expert panel** | 2 | Criteria for the selection of experts and transparent information on recruitment of the expert panel, socio-demographic details including information on expertise regarding the topic in question, (non)response and response rates over the ongoing iterations should be reported | 10 |
| **Description of the methods** | 3 | The methods employed need to be comprehensible; this includes information on preparatory steps (How was available evidence on the topic in question synthesized?), piloting of material and survey instruments, design of the survey instrument(s), the number and design of survey rounds, methods of data analysis, processing and synthesis of experts’ responses to inform the subsequent survey round and methodological decisions taken by the research team throughout the process | 9-12  See part I of the publication |
| **Procedure** | 4 | Flow chart to illustrate the stages of the Delphi process, including a preparatory phase, the actual ‘Delphi rounds’, interim steps of data processing and analysis, and concluding steps | See figure X part I of the publication  10-12 (part II) |
| **Definition and attainment of consensus** | 5 | It needs to be comprehensible to the reader how consensus was achieved throughout the process, including strategies to deal with non-consensus | 11-12 |
| **Results** | 6 | Reporting of results for each round separately is highly advisable in order to make the evolving of consensus over the rounds transparent. This includes figures showing the average group response, changes between rounds, as well as any modifications of the survey instrument such as deletion, addition or modification of survey items based on previous rounds | 12  Table 1 and 2 of part II  25, figures 1,2, 3, and 4 of part II |
| **Discussion of limitations** | 7 | Reporting should include a critical reflection of potential limitations and their impact of the resulting guidance | 25-28 |
| **Adequacy of conclusions** | 8 | The conclusions should adequately reflect the outcomes of the Delphi study with a view to the scope and applicability of the resulting practice guidance | 27-28 |
